# Supplementary material for: Efficacy, cost-minimization, and budget impact of a personalized discharge letter for basal cell carcinoma patients to reduce low-value follow-up care
Source: PLoS One. 2022 Jan 24;17(1):e0260978. doi: 10.1371/journal.pone.0260978 (PMC8786164; doi:10.1371/journal.pone.0260978)
Supplement: S1 File — (DOCX) [file pone.0260978.s001.docx]

**S1 File**

**Appendix S1:** Template personalized discharge letter, the parts in red are personalized

**Appendix S2:** Costs used in the cost-effectiveness analysis

**Appendix S3:** Probabilities and utilities for the CEA

**Appendix S4:** Scenario analysis software costs

**Appendix S5:** Scenario analysis budget impact uptake

**Appendix S1: Template personalized discharge letter, the parts in red are personalized**

Dear sir/madam patient,

We recently diagnosed you with a basal cell carcinoma on your face. Basal cell carcinoma is a form of skin cancer that grows slowly, hardly ever metastases and is typically easy to treat. Your (resident) dermatologist dr. Doctor treated this skin abnormality on dd-mm-yyyy. We are happy to provide you with personal information regarding your type of skin cancer.

**Result of your treatment with 5-Fluorouracil (Efudix) cream**
Your dermatologist has diagnosed you with a superficial basal cell carcinoma. Superficial means that the skin condition grows superficially. You have treated this basal cell carcinoma with Efudix cream. This is a substance that inhibits cell growth. The cancer cells in your skin absorb this substance, and as a result these cells can no longer grow and divide. During the treatment you probably suffered from skin irritation. This is due to the proper functioning of the cream. After the treatment this will disappear. This treatment rarely leaves a scar. A basal cell carcinoma treated with Efudix cream returns within 5 years in 10% of cases.

**Your chance of a subsequent basal cell carcinoma**
The chance of developing a new basal cell carcinoma at a different place on the body is generally higher if someone has had a basal cell carcinoma before. The more often skin cancer has occurred, the greater the chance of developing new spots. This was your first basal cell carcinoma. Based on your personal data, we will make an estimate of the chance that you will be able to develop a new basal cell carcinoma. We take into account your gender (male or female), age, lifestyle and the characteristics of the treated basal cell carcinoma. It is our expectation that the chance that you will get another basal cell carcinoma in the next 3 years is about 10-15%.

A basal cell carcinoma is usually not severe, but we want to prevent it from growing. We prefer to treat as early as possible. We therefore advise you to check your skin for suspicious spots in the future.

**Protect your skin well**
UV radiation from the sun and sunbeds increases the risk of developing basal cell carcinoma. The more often a person has been sunburned and the more exposed they are to the sun, the greater the risk of developing skin cancer later in life. This is why it is important to protect your skin from the sun. Use a sunscreen with a minimum factor 30. And wear clothing that protects you from the sun. Above all, look for the shade.

The sensitivity of your skin to sunlight depends, among other things, on your skin type. This is hereditary. We distinguish 6 different skin types. Skin types I, II and III are the most sensitive to UV radiation. The sun causes faster damage to the skin of these skin types than people with skin types IV, V or VI. That is why it is especially important for these skin types to protect the skin against UV radiation. But also with skin types IV, V, VI, it remains important to properly protect the skin against UV radiation.

We have determined skin type II for you.

| 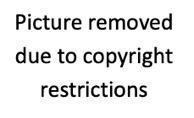 **Skintype I** | 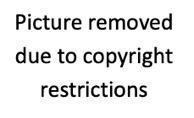**Skintype II** | 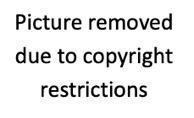**Skintype III** | 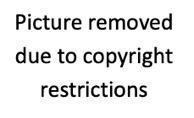**Skintype IV** | 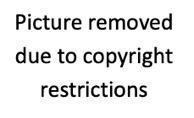**Skintype V** | 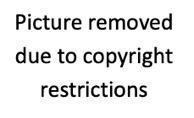**Skintype VI** |
| --- | --- | --- | --- | --- | --- |
| **Pale white** | **Fair white** | **Wite to olive** | **Olive to brown** | **Brown to dark brown** | **Dark brown to black** |
| **Red hair** | **Blond hair** | **Brown hair** | **Dark hair** | **Black hair** | **Black hair** |
|  |  |  |  |  |  |

**Follow-up of low-risk basal cell carcinoma**You have been successfully treated for a first basal cell carcinoma. Your dermatologist has checked and discussed the result of the treatment with you. You have a small chance of developing a new basal cell carcinoma and a potential recurrence of your current treated basal cell carcinoma is expected to give you little problems. You therefore fall into the category 'low-risk'. You do not have to stay under our regular follow-up. If you like, you can make a check-up appointment with your GP in a year's time. The doctor can then check your entire skin. We advise you to check your own skin as well. In the attached leaflet 'self-examination of your skin after basal cell carcinoma' you can read how to do that. This leaflet is also available online at *www.erasmusmc.nl/patientenfolders* under the specialty dermatology (oncology). More information can be found on the website of the Dutch Society of Dermatology and Venereology (*www.nvdv.nl*).

Will you discover a suspicious skin defect in the future? Then you can always contact your general practitioner.

Kind regards,

Dr. Doctor

(Resident) Dermatologist

*You take part in a scientific study on basal cell carcinoma at Erasmus MC in Rotterdam. This personal letter is part of this study.*

**Appendix S2: Costs used in the cost-effectiveness analysis**

Cost categories
To decide which costs are relevant for a cost-effectiveness analysis, one first has to decide on a perspective. The recommended perspective in the Netherlands is the societal[1]. The societal perspective requires information on costs of the following three categories: medical costs, costs for patient and family, and costs for other sectors[2].

*Medical costs*

Medical costs consist of direct costs related to the condition that is being studied. In this case, these are all the costs that are relevant to BCC follow-up care. It should also include all indirect medical costs that are created due to life years gained[2]. However, this intervention does not seek to lengthen the lives of BCC patients. It strives to lower the healthcare costs while maintaining the same quality of care. Therefore, the indirect medical costs will not be included in this analysis.
Costs can be measured and valued in two varieties: micro costing and gross costing. The choice impacts the accuracy of the cost estimates[3, 4].

To decide which type of costing is preferable, the Dutch BCC guideline was used to determine what a follow-up session at a specialist for BCC patients should contain. The guideline states that low-risk BCC patients should receive one follow-up visit within 6-12 months after treatment. A follow-up session should contain these two elements: inspection of the scar tissue and a full check of the skin. It is recommended that dermatologists inform their patients on the probability of recurrence and provide a brochure with information. It is also recommended to instruct patients on self-examination. Finally, the dermatologist should send the GP of the patient an update regarding the diagnosis and treatment[5]. A follow-up session at a GP is rather similar. GPs check the scar tissue and the skin of the patient once after treatment. They also advise patients on self-examination[6].

The aim of this intervention is to spread the innovation through the entire country when proven to be cost-effective. The costs used in the analysis should be generalizable for most dermatology departments and GP practices. Therefore, top-down gross costing is the best option for this analysis.
There are also medical costs created when one uses the intervention. Using the personalized discharge letter requires extra effort from a healthcare worker. On average it took about 2 minutes to develop and print the letter during the study. The letter has a standard format in which small alterations can be made to make it personal. These alterations include the name of the patient, the subtype of BCC, the location of the BCC, the type of treatment that was used, the name of the dermatologist and the prognosis. The price for the development was calculated by taking the average salary of dermatologists (all tiers) and dividing it into 2 minutes.

To make the letter available electronically, some software adjustments have to be made to existing electronic patient files. This requires one adjustment for each system. This article targeted the company with the largest market share. An estimate of €5,000 was made for the development and access for one hospital. Any extra hospital that wants to participate has to pay an estimated €1,000 to get access to the software add-on. The fee for the software only has to be paid once whereas the actual personalization of the letter comes with costs for each letter.

There is no training required for the creation of the letter. The current software systems that are used in the Netherlands are already capable of creating letters. This is adjustment is simply a new template that has to become available for all dermatologists. The actual making of the letter by a dermatologist does not differ from the other letters that can be created with the software program. Hence, there are no costs added for training.

*Costs for patient and family*

The questionnaires that were completed by the patients did not contain questions regarding their travel expenses, the type of transport they used or the distance they had to travel. National averages were used to estimate the costs for travel. The follow-up visit is a very general procedure that can be conducted in every healthcare center by a dermatologist. The expected travel costs are therefore low. In the Netherlands, the average distance to a hospital is considered to be 7.0 kilometers[2]. The type of transport is unknown. When the information is unavailable it is recommended to use the standard tariff for car and public transport since this is most often used. The standard tariff is €0.19 per kilometer[2]. The distance of 7.0 km has to be doubled since patients travel this distance twice; from their home to the hospital and back to their home.
 *Costs for other sectors*

When patients have to be present during a follow-up visit, they cannot use that time to work. This is known as productivity loss. There can be productivity loss for both paid and unpaid work. Loss of productivity was measured during the trial with the SF-HLQ questionnaire. This questionnaire is considered a reliable alternative of general absence registrations[7]. There are average costs for productivity available. The productivity cost for one hour of paid work differs for men and women. On average, the work of men results in €37,90 per hour. The cost for women is €31,60 per hour. There is no sex difference in costs for unpaid work. The price for this type of labor is €14,00 an hour. These prices are based on the average salary between the ages of 18 to 65[2].

Price index
The used reference prices date from several different years. The prices were indexed to 2019 using the Dutch derived consumer index prices and rounded to two decimals[8]. The 2019 price of older data was calculated by the following formula[2]:

$$reference price=older price*price index$$

Discounting
Since the model calculates results for ten years, both costs and health effects have to be discounted to present values. The Dutch guidelines recommend differential discounting with 4% for costs and 1,5% for health effects[1, 9]. The following formula was used to calculate the discounted prices[2]:

$$\sum_{t=0}^{n} K_{t} {(1+i)}^{-t}$$

In which K_t_ are the costs in year *t* and *i* is the discounting percentage.

Fixed prices
The entire cost-effectiveness analysis incorporates an uncertainty analysis of its outcomes. However, the input data on costs can also contain uncertainty. This study uses reference prices. These are considered to be fixed, are nationally assigned, and provided via the Dutch costing manual[2]. Therefore, they are free of uncertainty.

The costs for a follow-up session at a dermatologist were calculated by Vektis[10]. Vektis has access to all claims made by Dutch citizens at their health care insurers. This data provides insight in the average cost of a follow-up session for BCC patients after their first treatment. This number was derived from all Dutch institutions performing these follow-up sessions. This leaves no uncertainty regarding the price of a follow-up session.

The software costs were an estimation made by an IT manager. The actual costs for the software could not be obtained as this would be a tailored project. To correct for uncertainty in this estimation, a scenario analysis was performed. In this analysis the model was rerun with different implementation scenarios to ascertain its effect on the outcomes. One scenario was a local scenario, which consisted of implementation in one hospital. According to hospital declaration data in 2018, approximately 1,000 new low risk BCC patients were treated at the Erasmus MC. Dividing the €5,000 over these patients resulted in €5.00 software costs. The other scenario is a national implementation plan where the price of the software was divided over the number of patients who will benefit from the software update. Of the 77 Dutch hospitals, 50 are currently using Chipsoft’s HiX system, which is 65%[11]. About 48,000 individuals are diagnosed with a BCC every year. 65% of the incidence results in 31,169 individuals. Of these, 50% is considered to be low risk resulting in 15,585 individuals who can benefit from this software investment. Dividing the investment over the end users resulted in €3,47 investment per patient.

Calculations
The calculations shown in Table S2.1 show the average costs per patient associated with each visit and the indexed price for 2019.

**Table S2.1:** Calculations of the costs used in the cost-effectiveness analysis

| **Medical costs** | | **Indexed price** | **Calculations** |
| --- | --- | --- | --- |
| Follow-up session SP [10]  *Check of scar and skin*  *Providing information to patient*  *Update GP* | €112.69 | **€117.92** | €112.69*1.0464 |
| Follow-up session GP [2]  *Check of scar and skin*  *Providing information to patient* | €33.00 | **€34.45** | €33.00*1.0464/1.0025 |
| Intervention  *Development of letter [12]*    *Software adjustment*  *(Additional software access)*  *Software costs per patient* | €1.58  €5,000.00  (€1,000.00) | **€1.61**  **€5,000.00**  **(€1,000.00)**  **€3.47** | (((34.03+38.35+42.91+47.49+52.07+56.66+61.24)/7)/30)*1.0464/1.0301  (5,000+(49*1,000))/15,585 |
| **Total medical costs** | | €157.45 | |
| **Costs for patient and family** | |  |  |
| Travel expenses [2]  *7,0 km from home to hospital* | €2.66 | **€2.78** | ((€0.19*7.0)*2)*1.0464/1.0025 |
| **Total costs for patient and family** | | €2.78 | |
| **Costs for other sectors** | |  |  |
| Productivity loss paid work [2]  *Man*  *Woman* | €37.90  €31.60 | **€39.56**  **€32.98** | €37.90*1.0464/1.0025  €31.60*1.0464/1.0025 |
| Productivity loss unpaid work [2] | €14.00 | **€14.61** | €14.00*1.0464/1.0025 |
| **Total costs for other sectors** | | €87.15 | |

Abbreviations: SP, Medical specialist; GP, General practitioner.

**Appendix S3: Probabilities and utilities for the CEA**

Probability of getting a new skin cancer diagnosis
The probabilities were derived from literature and are listed in Table S3.1[13].

**Table S3.1:** Probability of new skin cancer diagnosis

|  | **BCC after BCC** | **cSCC after BCC** | **Melanoma after BCC** |
| --- | --- | --- | --- |
| First BCC | 0.258 | 0.045 | 0.004 |

Abbreviations: BCC, Basal cell carcinoma; cSCC, Cutaneous squamous cell carcinoma

These chances of getting a new skin cancer diagnosis are based on the assumption that the intervention (i.e., reducing the number of follow-up visits) has no effect on the chance of developing new tumors or mortality. This effect has recently been studied in melanoma patients where the number of follow-up sessions was lowered which was deemed as safe and cost-effective[14]. This model uses the outcomes of the melanoma study since there is no information available on BCC and cSCC patients as of yet.

Probability of making a control appointment
The probability of making a follow-up visit differ with each type of follow-up. The probabilities are divided into the control and intervention group.

 *Control group*

The behavior of 88 first time BCC patients was monitored for five years in a prior Erasmus MC study[15]. The probabilities in Table S3.2 are of patients who had a low-risk BCC and no recurrence during these five years. They received standard care and were not exposed to any interventions. These results were used to model the appointments from year two onwards. The results from the first year stem from the data of this trial. The number of appointments stabilizes after five years. Since the time horizon of the model is set to ten years, the assumption was made that the chance of a patient making an appointment does not change after year five.

**Table S3.2:** Overview appointments control

| **Year** | **Probability of making an appointment** |
| --- | --- |
| **First year**  *No appointment*  *1 appointment*  *2 appointments*  *3 appointments*  *4 appointments*  *5 appointments*  *6 appointments*  *7-10 appointments*  *>10 appointments* | 0.138  0.325  0.375  0.136  0.018  0.006  0.002  0.000  0.000 |
| **Second year**  *No appointment*  *1 appointment*  *2 appointments*  *3-5 appointments*  *6-10 appointments*  *>10 appointments* | 0.70  0.21  0.04  0.04  0.01  0.00 |
| **Third year**  *No appointment*  *1 appointment*  *2 appointments*  *3-5 appointments*  *6-10 appointments*  *>10 appointments* | 0.82  0.12  0.05  0.01  0.00  0.00 |
| **Fourth year**  *No appointment*  *1 appointment*  *2 appointments*  *3-5 appointments*  *6-10 appointments*  *>10 appointments* | 0.78  0.16  0.00  0.05  0.00  0.00 |
| **Fifth year**  *No appointment*  *1 appointment*  *2 appointments*  *3-5 appointments*  *6-10 appointments*  *>10 appointments* | 0.94  0.03  0.00  0.03  0.00  0.00 |

*Intervention group*

The behavior of first time BCC patients who received a personalized letter was monitored for 1 year during the trial. Patients recorded the number of visits they made to the GP or the specialist for their BCC in the surveys. The probabilities are presented in Table S3.3. The probabilities of the control cohort were applied to the intervention cohort from year two onwards.

**Table S3.3:** Overview appointments intervention

| **Year** | **Probability of making an appointment** |
| --- | --- |
| **First year**  *No appointment*  *1 appointment*  *2 appointments*  *3 appointments*  *4 appointments*  *5 appointments*  *6 appointments*  *7-10 appointments*  *>10 appointments* | 0.209  0.380  0.266  0.114  0.023  0.006  0.002  0.000  0.000 |

Utilities
The utilities for no new tumor and a new BCC were calculated from the EQ-5D-3L using the Dutch tariff[16]. The calculated utilities are higher than average national scores[17]. The EQ-5D-3L is known to be less detailed than the EQ-5D-5L, which explains the small impact minor changes have on the overall score[18].

The number of patients who were diagnosed with a melanoma or a cSCC during this study was too low to use the outcomes of the EQ-5D-3L. Two patients were diagnosed with a melanoma and three with a cSCC. Melanoma scores were substituted with results from literature. There is no study that clearly stated the quality of life of cSCC patients alone. Studies frequently combine BCCs and cSCCs when calculating the quality of life for these patients[19-21]. The Global Burden of Disease Study gave both types of keratinocyte cancer the same burden of disease with identical disability weights[22]. Therefore, this model assumes that the impact of a cSCC on a patients’ quality of life is the same as a BCC. The outcomes of the EQ-5D-3L for BCCs were used for cSCCs as well. The utilities for every health state overall are expressed in Table S3.4.

**Table S3.4:** Overview utilities

| **Type** | **Mean utility** | **SD** | **SE** |
| --- | --- | --- | --- |
| Melanoma [23] | 0.719 | 0.211 | 0.011 |
| BCC | 0.910 | 0.113 | 0.025 |
| cSCC | 0.910 | 0.113 | 0.025 |
| None | 0.910 | 0.165 | 0.010 |

Abbreviations: BCC, basal cell carcinoma; cSCC, cutaneous squamous cell carcinoma; SD, standard deviation; SE, standard error.

Mortality risks
Table S3.5 specifies the annual probabilities of dying per age and gender based on observed mortality rates in 2018 in the Netherlands[24].

**Table S3.5:** Overview mortality

| Gender | Age (on 31^st^ of December) | Probability of dying |
| --- | --- | --- |
| Men | 18 | 0.00022 |
| Men | 19 | 0.00032 |
| Men | 20 | 0.00042 |
| Men | 21 | 0.00029 |
| Men | 22 | 0.00045 |
| Men | 23 | 0.00030 |
| Men | 24 | 0.00037 |
| Men | 25 | 0.00046 |
| Men | 26 | 0.00040 |
| Men | 27 | 0.00037 |
| Men | 28 | 0.00057 |
| Men | 29 | 0.00046 |
| Men | 30 | 0.00049 |
| Men | 31 | 0.00065 |
| Men | 32 | 0.00048 |
| Men | 33 | 0.00054 |
| Men | 34 | 0.00050 |
| Men | 35 | 0.00066 |
| Men | 36 | 0.00062 |
| Men | 37 | 0.00082 |
| Men | 38 | 0.00073 |
| Men | 39 | 0.00077 |
| Men | 40 | 0.00074 |
| Men | 41 | 0.00095 |
| Men | 42 | 0.00104 |
| Men | 43 | 0.00116 |
| Men | 44 | 0.00122 |
| Men | 45 | 0.00151 |
| Men | 46 | 0.00132 |
| Men | 47 | 0.00173 |
| Men | 48 | 0.00208 |
| Men | 49 | 0.00202 |
| Men | 50 | 0.00186 |
| Men | 51 | 0.00252 |
| Men | 52 | 0.00292 |
| Men | 53 | 0.00279 |
| Men | 54 | 0.00357 |
| Men | 55 | 0.00378 |
| Men | 56 | 0.00400 |
| Men | 57 | 0.00491 |
| Men | 58 | 0.00538 |
| Men | 59 | 0.00576 |
| Men | 60 | 0.00666 |
| Men | 61 | 0.00715 |
| Men | 62 | 0.00804 |
| Men | 63 | 0.00902 |
| Men | 64 | 0.00985 |
| Men | 65 | 0.01086 |
| Men | 66 | 0.01208 |
| Men | 67 | 0.01315 |
| Men | 68 | 0.01421 |
| Men | 69 | 0.01565 |
| Men | 70 | 0.01724 |
| Men | 71 | 0.01852 |
| Men | 72 | 0.02163 |
| Men | 73 | 0.02399 |
| Men | 74 | 0.02651 |
| Men | 75 | 0.02925 |
| Men | 76 | 0.03180 |
| Men | 77 | 0.03616 |
| Men | 78 | 0.03881 |
| Men | 79 | 0.04482 |
| Men | 80 | 0.05199 |
| Men | 81 | 0.05526 |
| Men | 82 | 0.06791 |
| Men | 83 | 0.07365 |
| Men | 84 | 0.08305 |
| Men | 85 | 0.09376 |
| Men | 86 | 0.11164 |
| Men | 87 | 0.12279 |
| Men | 88 | 0.14195 |
| Men | 89 | 0.15732 |
| Men | 90 | 0.18123 |
| Men | 91 | 0.19571 |
| Men | 92 | 0.21295 |
| Men | 93 | 0.23114 |
| Men | 94 | 0.26255 |
| Men | 95 | 0.28567 |
| Men | 96 | 0.31253 |
| Men | 97 | 0.31726 |
| Men | 98 | 0.33859 |
| Men | 99 or older | 0.36676 |
| Women | 18 | 0.00022 |
| Women | 19 | 0.00011 |
| Women | 20 | 0.00020 |
| Women | 21 | 0.00020 |
| Women | 22 | 0.00013 |
| Women | 23 | 0.00015 |
| Women | 24 | 0.00018 |
| Women | 25 | 0.00015 |
| Women | 26 | 0.00025 |
| Women | 27 | 0.00022 |
| Women | 28 | 0.00017 |
| Women | 29 | 0.00030 |
| Women | 30 | 0.00027 |
| Women | 31 | 0.00025 |
| Women | 32 | 0.00045 |
| Women | 33 | 0.00030 |
| Women | 34 | 0.00030 |
| Women | 35 | 0.00042 |
| Women | 36 | 0.00038 |
| Women | 37 | 0.00037 |
| Women | 38 | 0.00046 |
| Women | 39 | 0.00056 |
| Women | 40 | 0.00059 |
| Women | 41 | 0.00057 |
| Women | 42 | 0.00089 |
| Women | 43 | 0.00073 |
| Women | 44 | 0.00102 |
| Women | 45 | 0.00095 |
| Women | 46 | 0.00121 |
| Women | 47 | 0.00126 |
| Women | 48 | 0.00126 |
| Women | 49 | 0.00140 |
| Women | 50 | 0.00169 |
| Women | 51 | 0.00191 |
| Women | 52 | 0.00222 |
| Women | 53 | 0.00227 |
| Women | 54 | 0.00254 |
| Women | 55 | 0.00314 |
| Women | 56 | 0.00312 |
| Women | 57 | 0.00387 |
| Women | 58 | 0.00403 |
| Women | 59 | 0.00448 |
| Women | 60 | 0.00506 |
| Women | 61 | 0.00550 |
| Women | 62 | 0.00603 |
| Women | 63 | 0.00660 |
| Women | 64 | 0.00675 |
| Women | 65 | 0.00789 |
| Women | 66 | 0.00841 |
| Women | 67 | 0.00860 |
| Women | 68 | 0.00927 |
| Women | 69 | 0.00988 |
| Women | 70 | 0.01174 |
| Women | 71 | 0.01281 |
| Women | 72 | 0.01403 |
| Women | 73 | 0.01630 |
| Women | 74 | 0.01794 |
| Women | 75 | 0.01864 |
| Women | 76 | 0.02202 |
| Women | 77 | 0.02405 |
| Women | 78 | 0.02777 |
| Women | 79 | 0.02993 |
| Women | 80 | 0.03413 |
| Women | 81 | 0.03873 |
| Women | 82 | 0.04460 |
| Women | 83 | 0.05318 |
| Women | 84 | 0.05872 |
| Women | 85 | 0.06952 |
| Women | 86 | 0.08159 |
| Women | 87 | 0.09371 |
| Women | 88 | 0.10502 |
| Women | 89 | 0.12540 |
| Women | 90 | 0.13453 |
| Women | 91 | 0.15635 |
| Women | 92 | 0.17848 |
| Women | 93 | 0.20248 |
| Women | 94 | 0.22220 |
| Women | 95 | 0.24917 |
| Women | 96 | 0.26724 |
| Women | 97 | 0.30390 |
| Women | 98 | 0.31418 |
| Women | 99 or older | 0.37089 |

The Table S3.6 specifies the disease specific mortality probabilities associated with BCC, cSCC and melanoma. These rates are under the assumption that new carcinomas are discovered in a relatively early stage and that there are no competing risks which can increase the mortality risk of skin cancer.

**Table S3.6:** Disease specific mortality of skin cancer[25-27]

| **Diagnosis** | **Mortality probabilities** |
| --- | --- |
| Basal Cell Carcinoma | 0.001 |
| Squamous Cell Carcinoma | 0.021 |
| Melanoma | 0.071 |

**Appendix 4: Scenario analysis software costs**

To monitor the effect of different software implementation on the model’s outcome, the model has been rerun with software prices per patient of €5.00 and €3.47. Table S4.1 shows the results of the original analysis as presented in the main text. In Figure S4.1 one can see the incremental cost-effectiveness plane with the outcomes after five years.

**Table S4.1:** Original estimate local implementation

| Probabilistic results for five years | | Probabilistic results for ten years | |
| --- | --- | --- | --- |
| Expected costs intervention | €350.24 | Expected costs intervention | €391.77 |
| Expected costs control | €373.17 | Expected costs control | €414.69 |
| Expected QALYs intervention | 4.123 | Expected QALYs intervention | 7.249 |
| Expected QALYs control | 4.120 | Expected QALYs control | 7.241 |
| Expected incremental costs | - €22.94 | Expected incremental costs | - €22.92 |
| Expected incremental QALYs | <0.003 | Expected incremental QALYs | >0,009 |

**Figure S4.1: Incremental cost-effectiveness plane for 5 years**

Table S4.2 shows the outcomes of the model when the software price can be divided nationally over all eligible patients that are treated in the healthcare centers who use the required electronic patient files. Figure S4.2 shows the incremental cost-effectiveness plane for five years.

**Table S4.2:** Scenario 2 national implementation

| Probabilistic results for five years | | Probabilistic results for ten years | |
| --- | --- | --- | --- |
| Expected costs intervention | €348.70 | Expected costs intervention | €390.37 |
| Expected costs control | €373.16 | Expected costs control | €414.79 |
| Expected QALYs intervention | 4.124 | Expected QALYs intervention | 7.251 |
| Expected QALYs control | 4.121 | Expected QALYs control | 7.242 |
| Expected incremental costs | - €24.45 | Expected incremental costs | - €24.41 |
| Expected incremental QALYs | <0.003 | Expected incremental QALYs | <0.009 |

**Figure S4.2:** Incremental cost-effectiveness plane for 5 years

If the software costs are divided over a national population the five-year cost savings would be €1.51 higher per patient. In ten years, the cost saving would be €1.49 higher. This is a 6.6% and a 6.5% increase in savings. Increasing the number of patients who participate in the intervention affects has minimal effects on the cost savings per patient.

**Appendix S5: Scenario analysis budget impact uptake**

The uptake of the intervention has an impact on the cost savings that result from it. The more patients participate, the higher the cost savings. The budget impact is calculated for different levels of uptake, the cost categories and the implementation setting.

**Table S5.1:** Budget impact analysis for local implementation

| Year | 2021 | 2022 | 2023 | 2024 | 2025 | Total |
| --- | --- | --- | --- | --- | --- | --- |
| Local implementation | | | | | | |
| Eligible population | 1,260 | 1,361 | 1,470 | 1,587 | 1,714 | 7,392 |
| Expected uptake | 40% | 50% | 60% | 75% | 75% | 75% |
| Patients receiving the intervention | 504 | 680 | 882 | 1,190 | 1286 | 4,542 |
| Medical costs | -€9,329 | -€12,594 | -€16,322 | -€22,035 | -€23,798 | -€84,080 |
| Costs for patients | -€277 | -€375 | -€485 | -€655 | -€708 | -€2,501 |
| Productivity loss | -€1,954 | -€2,638 | -€3,418 | -€4,615 | -€4,984 | -€17,608 |
| Budget impact | -€11,560 | -€15,607 | -€20,226 | -€27,305 | -€29,490 | -€104,188 |
| *Lower estimate* | -€5,780 | -€9,364 | -€13,484 | -€18,203 | -€19,659 | -€66,491 |
| *Higher estimate* | -€14,451 | -€18,728 | -€26,968 | -€29,126 | -€31,456 | -€120,728 |

The original uptake in the local setting estimates resulted in a cost saving of €104,188 after five years (Table S5.1). Lowering the uptake, where after five years only 50% of the eligible population receives the interventions created a cost saving of €66,491. A high uptake of the intervention saved €120,728. Lowering the uptakes drops the cost saving by 36%. A higher uptake increases the saving by 16%.

**Table S5.2:** Budget impact analysis for national implementation

| Year | 2021 | 2022 | 2023 | 2024 | 2025 | Total |
| --- | --- | --- | --- | --- | --- | --- |
| National implementation | | | | | | |
| Eligible population | 30,247 | 32,667 | 35,280 | 38,103 | 41,151 | 177,448 |
| Expected uptake | 40% | 50% | 60% | 75% | 75% | 75% |
| Patients receiving the intervention | 12,099 | 16,333 | 21,168 | 28,577 | 30,863 | 109,040 |
| Medical costs | -€243,866 | -€329,219 | -€426,668 | -€576,002 | -€622,082 | -€2,197,836 |
| Costs for patients | -€6,931 | -€9,357 | -€12,126 | -€16,371 | -€17,680 | -€62,465 |
| Productivity loss | -€45,055 | -€60,825 | -€78,829 | -€106,419 | -€114,932 | -€406,060 |
| Budget impact | -€295,852 | -€399,400 | -€517,623 | -€698,791 | -€754,694 | -€2,666,361 |
| *Lower estimate* | -€147,926 | -€239,640 | -€345,082 | -€465,861 | -€503,129 | -€1,701,638 |
| *Higher estimate* | -€369,815 | -€479,280 | -€690,164 | -€745,377 | -€805,007 | -€3,089,644 |

In the national implementation the expected budget impact was -€2,666,361 (Table S5.2). With a lower uptake it resulted in €1,701,638 cost savings. The higher uptake was €3,089,644 of cost savings. A lower uptake reduced the budget impact with 36%. A higher uptake increased the budget impact with 16%.

**References**

13. Deckers EA, Hoekstra-Weebers J, Damude S, et al. The MELFO Study: A Multicenter, Prospective, Randomized Clinical Trial on the Effects of a Reduced Stage-Adjusted Follow-Up Schedule on Cutaneous Melanoma IB-IIC Patients-Results After 3 Years. *Ann Surg Oncol.* 2020;27(5):1407-1417.

14. Venerologie NVvDe. Richtlijn Basaalcelcarcinoom - Follow-up. 2016; <https://richtlijnendatabase.nl/richtlijn/basaalcelcarcinoom/follow-up_bcc.html#tab-content-starting-question>. Accessed 19-02, 2020.

25. Nederland Z. *Richtlijn voor het uitvoeren van economische evaluaties in de gezondheidszorg.* Zorginstituut Nederland;2016.

27. de Vroome EMM, de Koppes LLJ, Smulders PGW, van den Bossche SNJ. Verzuimmeting via zelfrapportage en registratie: verschillen tussen de Nationale Enquête Arbeidsomstandigheden en de Nationale Verzuim Statistiek. *TSG.* 2010;88(2):71-78.

33. Statistiek CBvd. Consumentenprijzen; Prijsindex. In: Statistiek CBvd, ed: Centraal Bureau voor de Statistiek; 2020.

34. Attema AE, Brouwer WBF, Claxton K. Discounting in Economic Evaluations. *Pharmacoeconomics.* 2018;36(7):745-758.

35. Flohil S. Data bezoeken BCC PCC 5 jaar follow-up. Rotterdam: Erasmus Medisch Centrum; 2017:1-4.

38. Hakkaart - van Roijen L, van der Linden N, Bouwmans C, Kanters T, Swan Tan S. *Kostenhandleiding:  Methodologie van kostenonderzoek en  referentieprijzen voor economische evaluaties  in de gezondheidszorg*Zorginstituut Nederland;2016.

40. Tromme I, Devleesschauwer B, Beutels P, et al. Health-related quality of life in patients with melanoma expressed as utilities and disability weights. *British Journal of Dermatology.* 2014;171(6):1443-1450.

41. Flohil SC, van der Leest RJT, Arends LR, de Vries E, Nijsten T. Risk of subsequent cutaneous malignancy in patients with prior keratinocyte carcinoma: A systematic review and meta-analysis. *European Journal of Cancer.* 2013;49(10):2365-2375.

42. Chinem VP, Miot HA. Epidemiology of basal cell carcinoma. *Anais Brasileiros de Dermatologia.* 2011;86(2):292-305.

43. Schmults CD, Karia PS, Carter JB, Han J, Qureshi AA. Factors Predictive of Recurrence and Death From Cutaneous Squamous Cell Carcinoma: A 10-Year, Single-Institution Cohort Study. *JAMA Dermatology.* 2013;149(5):541-547.

44. Shen W, Sakamoto N, Yang L. Melanoma-specific mortality and competing mortality in patients with non-metastatic malignant melanoma: a population-based analysis. *BMC Cancer.* 2016;16(1):413.

45. Statistiek CBvd. Levensverwachting. In: Statistiek CBvd, ed: Centraal Bureau voor de Statistiek; 2019.

46. Vektis. Cutane maligniteit -  29499. Vektis; 2015.

47. Ziekenhuizen NVv. *Salaristabellen Medisch Specialisten 2017-2019.* Nederlandse Vereniging van Ziekenhuizen;2017.

54. Genootschap NH. NHG-Standaard Verdachte Huidafwijkingen. 2017; <https://www.nhg.org/standaarden/volledig/nhg-standaard-verdachte-huidafwijkingen#idp448144>.

56. Špacírová Z, Epstein D, García-Mochón L, Rovira J, Olry de Labry Lima A, Espín J. A general framework for classifying costing methods for economic evaluation of health care. *European Journal of Health Economics.* 2020.

57. Tan SS, Rutten FFH, van Ineveld BM, Redekop WK, Hakkaart-van Roijen L. Comparing methodologies for the cost estimation of hospital services. *The European Journal of Health Economics.* 2009;10(1):39-45.

58. Bukman B. Het complete epd-overzicht: welk ziekenhuis heeft welke leverancier? *Zorgvisie* 2018; <https://www.zorgvisie.nl/epd-overzicht/>, 2020.

59. Lamers LM, McDonnell J, Stalmeier PFM, Krabbe PFM, Busschbach JJV. The Dutch tariff: results and arguments for an effective design for national EQ-5D valuation studies. *Health Economics.* 2006;15(10):1121-1132.

60. M. Versteegh M, M. Vermeulen K, M. A. A. Evers S, de Wit GA, Prenger R, A. Stolk E. Dutch Tariff for the Five-Level Version of EQ-5D. *Value in Health.* 2016;19(4):343-352.

61. Janssen MF, Bonsel GJ, Luo N. Is EQ-5D-5L Better Than EQ-5D-3L? A Head-to-Head Comparison of Descriptive Systems and Value Sets from Seven Countries. *PharmacoEconomics.* 2018;36(6):675-697.

62. Abedini R, Nasimi M, Noormohammad Pour P, Moghtadaie A, Tohidinik HR. Quality of Life in Patients with Non-melanoma Skin Cancer: Implications for Healthcare Education Services and Supports. *Journal of Cancer Education.* 2019;34(4):755-759.

63. Arts LPJ, Waalboer-Spuij R, de Roos K-P, et al. Health-Related Quality of Life, Satisfaction with Care, and Cosmetic Results in Relation to Treatment among Patients with Keratinocyte Cancer in the Head and Neck Area: Results from the PROFILES Registry. *Dermatology (Basel, Switzerland).* 2020;236(2):133-142.

64. Chren M-M, Sahay AP, Bertenthal DS, Sen S, Seth Landefeld C. Quality-of-Life Outcomes of Treatments for Cutaneous Basal Cell Carcinoma and Squamous Cell Carcinoma. *Journal of Investigative Dermatology.* 2007;127(6):1351-1357.

65. James SL, Abate D, Abate KH, et al. Global, regional, and national incidence, prevalence, and years lived with disability for 354 Diseases and Injuries for 195 countries and territories, 1990-2017: A systematic analysis for the Global Burden of Disease Study 2017. *The Lancet.* 2018;392(10159):1789-1858.

1. Nederland Z. Richtlijn voor het uitvoeren van economische evaluaties in de gezondheidszorg. Zorginstituut Nederland, 2016.

2. Hakkaart - van Roijen L, van der Linden N, Bouwmans C, Kanters T, Swan Tan S. Kostenhandleiding:  Methodologie van kostenonderzoek en  referentieprijzen voor economische evaluaties  in de gezondheidszorg Zorginstituut Nederland, 2016.

3. Špacírová Z, Epstein D, García-Mochón L, Rovira J, Olry de Labry Lima A, Espín J. A general framework for classifying costing methods for economic evaluation of health care. European Journal of Health Economics. 2020. doi: 10.1007/s10198-019-01157-9.

4. Tan SS, Rutten FFH, van Ineveld BM, Redekop WK, Hakkaart-van Roijen L. Comparing methodologies for the cost estimation of hospital services. The European Journal of Health Economics. 2009;10(1):39-45. doi: 10.1007/s10198-008-0101-x.

5. Venerologie NVvDe. Richtlijn Basaalcelcarcinoom - Follow-up: Nederlandse Vereniging voor Dermatologie en Venerologie; 2016 [cited 2020 19-02]. Available from: <https://richtlijnendatabase.nl/richtlijn/basaalcelcarcinoom/follow-up_bcc.html#tab-content-starting-question>.

6. Genootschap NH. NHG-Standaard Verdachte Huidafwijkingen 2017. Available from: <https://www.nhg.org/standaarden/volledig/nhg-standaard-verdachte-huidafwijkingen#idp448144>.

7. de Vroome EMM, de Koppes LLJ, Smulders PGW, van den Bossche SNJ. Verzuimmeting via zelfrapportage en registratie: verschillen tussen de Nationale Enquête Arbeidsomstandigheden en de Nationale Verzuim Statistiek. TSG. 2010;88(2):71-8. doi: 10.1007/BF03089552.

8. Statistiek CBvd. Consumentenprijzen; Prijsindex. In: Statistiek CBvd, editor.: Centraal Bureau voor de Statistiek; 2020.

9. Attema AE, Brouwer WBF, Claxton K. Discounting in Economic Evaluations. PharmacoEconomics. 2018;36(7):745-58. doi: 10.1007/s40273-018-0672-z.

10. Vektis. Cutane maligniteit -  29499. Vektis; 2015.

11. Bukman B. Het complete epd-overzicht: welk ziekenhuis heeft welke leverancier? : Bohn Stafleu van Loghum; 2018 [cited 2020]. Available from: <https://www.zorgvisie.nl/epd-overzicht/>.

12. Ziekenhuizen NVv. Salaristabellen Medisch Specialisten 2017-2019. Nederlandse Vereniging van Ziekenhuizen, 2017.

13. Flohil SC, van der Leest RJT, Arends LR, de Vries E, Nijsten T. Risk of subsequent cutaneous malignancy in patients with prior keratinocyte carcinoma: A systematic review and meta-analysis. European Journal of Cancer. 2013;49(10):2365-75. doi: <https://doi.org/10.1016/j.ejca.2013.03.010>.

14. Deckers EA, Hoekstra-Weebers J, Damude S, Francken AB, Ter Meulen S, Bastiaannet E, et al. The MELFO Study: A Multicenter, Prospective, Randomized Clinical Trial on the Effects of a Reduced Stage-Adjusted Follow-Up Schedule on Cutaneous Melanoma IB-IIC Patients-Results After 3 Years. Ann Surg Oncol. 2020;27(5):1407-17. PubMed PMID: 31535302.

15. Flohil S. Data bezoeken BCC PCC 5 jaar follow-up. 2017.

16. Lamers LM, McDonnell J, Stalmeier PFM, Krabbe PFM, Busschbach JJV. The Dutch tariff: results and arguments for an effective design for national EQ-5D valuation studies. Health Economics. 2006;15(10):1121-32. doi: 10.1002/hec.1124.

17. M. Versteegh M, M. Vermeulen K, M. A. A. Evers S, de Wit GA, Prenger R, A. Stolk E. Dutch Tariff for the Five-Level Version of EQ-5D. Value in Health. 2016;19(4):343-52. doi: <https://doi.org/10.1016/j.jval.2016.01.003>.

18. Janssen MF, Bonsel GJ, Luo N. Is EQ-5D-5L Better Than EQ-5D-3L? A Head-to-Head Comparison of Descriptive Systems and Value Sets from Seven Countries. PharmacoEconomics. 2018;36(6):675-97. doi: 10.1007/s40273-018-0623-8. PubMed PMID: 29470821.

19. Abedini R, Nasimi M, Noormohammad Pour P, Moghtadaie A, Tohidinik HR. Quality of Life in Patients with Non-melanoma Skin Cancer: Implications for Healthcare Education Services and Supports. Journal of Cancer Education. 2019;34(4):755-9. doi: 10.1007/s13187-018-1368-y.

20. Arts LPJ, Waalboer-Spuij R, de Roos K-P, Thissen MRTM, Scheijmans LJ, Aarts MJ, et al. Health-Related Quality of Life, Satisfaction with Care, and Cosmetic Results in Relation to Treatment among Patients with Keratinocyte Cancer in the Head and Neck Area: Results from the PROFILES Registry. Dermatology (Basel, Switzerland). 2020;236(2):133-42. Epub 2019/08/21. doi: 10.1159/000502033. PubMed PMID: 31434078.

21. Chren M-M, Sahay AP, Bertenthal DS, Sen S, Seth Landefeld C. Quality-of-Life Outcomes of Treatments for Cutaneous Basal Cell Carcinoma and Squamous Cell Carcinoma. Journal of Investigative Dermatology. 2007;127(6):1351-7. doi: <https://doi.org/10.1038/sj.jid.5700740>.

22. James SL, Abate D, Abate KH, Abay SM, Abbafati C, Abbasi N, et al. Global, regional, and national incidence, prevalence, and years lived with disability for 354 Diseases and Injuries for 195 countries and territories, 1990-2017: A systematic analysis for the Global Burden of Disease Study 2017. The Lancet. 2018;392(10159):1789-858. doi: 10.1016/S0140-6736(18)32279-7.

23. Tromme I, Devleesschauwer B, Beutels P, Richez P, Leroy A, Baurain JF, et al. Health-related quality of life in patients with melanoma expressed as utilities and disability weights. British Journal of Dermatology. 2014;171(6):1443-50. doi: 10.1111/bjd.13262.

24. Statistiek CBvd. Levensverwachting. In: Statistiek CBvd, editor.: Centraal Bureau voor de Statistiek; 2019.

25. Chinem VP, Miot HA. Epidemiology of basal cell carcinoma. Anais Brasileiros de Dermatologia. 2011;86(2):292-305. doi: 10.1590/S0365-05962011000200013.

26. Schmults CD, Karia PS, Carter JB, Han J, Qureshi AA. Factors Predictive of Recurrence and Death From Cutaneous Squamous Cell Carcinoma: A 10-Year, Single-Institution Cohort Study. JAMA Dermatology. 2013;149(5):541-7. doi: 10.1001/jamadermatol.2013.2139.

27. Shen W, Sakamoto N, Yang L. Melanoma-specific mortality and competing mortality in patients with non-metastatic malignant melanoma: a population-based analysis. BMC Cancer. 2016;16(1):413. doi: 10.1186/s12885-016-2438-3.
