# Supplementary material for: Efficacy, cost-minimization, and budget impact of a personalized discharge letter for basal cell carcinoma patients to reduce low-value follow-up care
Source: PLoS One. 2022 Jan 24;17(1):e0260978. doi: 10.1371/journal.pone.0260978 (PMC8786164; doi:10.1371/journal.pone.0260978)
Supplement: Attachment — Submitted filename: Response to reviewers.docx [file pone.0260978.s002.docx]

Reviewer #1: Thank you for asking me to review this paper. This paper looks at the use of a personalised software generated discharge letter to reduce follow up visits following a first BCC.

1) Prevalence is misused as a term- 80% of Caucasians don’t have a BCC.

*The term was indeed misused and has been adjusted accordingly. (p.3, line 76)*

2) They don’t define a low risk BCC- or justify 50% of BCCs in their cohort being low risk.

*Low-risk BCCs are defined as* *primary BCC, < 2 cm, located outside the H-zone, with a nodular or superficial subtype according to the Dutch BCC guidelines. This has been added to the manuscript. (p.3, lines 86-87)*

*Regarding the justification of 50% of BCCs; This number was extrapolated from the previous work of the authors (de Vries et al.), as cited in the manuscript.*

3) They should be clear if their group pf BCCS were all completely excised and if so what parameters they use to define that – e.g mohs/ 1mm or more histological clearance at all margins on standard surgical excision with curative intent.

*We indeed do not provide the numbers of completely excised BCCs. This has not been registered for all patients. We acknowledge that this is a limitation of our study and have added it as such to the manuscript. (p.16, lines 354-355)*

4) Software related costs are mentioned- they should state this is one way of delivering this, but as with other patient letters, software is not mandatory.

*By software costs we intended the costs needed to implement the feature in the current electronic health record system the hospital already uses. We have clarified this in p.7, lines 198-199.*

5) The introduction of new guidelines could have had a significant impact on the behaviour of

and while the authors are right to mention is, it is a major risk that this led to some or most of the reduction in visits. They should state how follow up guidance changed to be clear on what the impact may have been

*We agree with the reviewer that clarifying this is valuable for the interpretation of the discussion. We have clarified in the discussion that this is the first skin cancer guideline for GPs (p.15, line 340) and that the guidelines states that after complete excision and a post-treatment evaluation after 3 months, further monitoring is not necessary (p.15, lines 343-344). However, we would like to emphasize that there have not been any changes in the dermatologist guidelines regarding BCC follow-up care, and the dermatologist is in control of the patients’ follow-up schedule (p16, lines 349-350)*

6) This paper demonstrates well that follow up was reduced coinciding with introduction of a personalised discharge letter. They explore that there is potentially a financial disincentive for clinicians to discharge a patient at one follow up visits. It is common practice in the UK for further visits to simply not be funded and it would be useful to discuss the pros and cons of excessive follow ups, from a patient and clinician and health care economy perspective.

*We agree with the reviewer that a further exploration on the effect of low-value care is an interesting direction. We have chosen to focus on the possible implementation effects of this particular intervention on all affected parties, ranging from employers to clinicians and insurers. We have added an extra perspective to the manuscript (p.15, line 335-337).*

Reviewer #2: Thank you for the nice report. It is nicely written. Correct use of statistics and well explained. The article adds value to current literature.

1) What is written in the personalized discharge letter? An example in het Supplementary would be useful. What factors in the letter make it 'personal'/ what are the differences between the letters and based on which characteristics of the patient?

I have no further comments.

*We are sorry to notice that there might have been an administrative issue during the submittal process. An example of the personalized letter was already added in the supplementary. We hope that this will find you well in the current rebuttal.*

**Editorial comments**

1) A key modelling assumption was that the introduction of personalized discharge letter had no effect on clinical outcomes and patient QALYs (i.e., the "low-value" follow-up visits were assumed to have zero clinical value in the model). Hence, it is recommended to discuss how evidence-based this assumption was. Given that no QALY difference was allowed across intervention and control cohorts by the model structure (except for stochastic difference in the probabilistic sensitivity analysis), the developed model is considered to be a cost minimization model, rather than cost-utility model: please consider adjusting the manuscript title accordingly.

*The effect of the personalized letter on the utility values was measured in the current study by administrating the EQ-5D-3L questionnaire. We agree with the editor that by having similar utility values in each group, the analysis is in fact a cost-minimization analysis. Extra clarification has been added to the manuscript (p. 7, line 180-182). We have also changed the title to “Efficacy, cost-minimization, and budget impact of a personalized discharge letter for basal cell carcinoma patients to reduce low-value follow-up care”*

2) For better reproducibility, please make an explicit statement about the applied appointment probabilities in Years 2-9 in the intervention cohort in Table S3.2.

*We have improved the readability by starting with the control group and providing extra specification about the probabilities in the supplementary material.*

3) In line with the comments of Reviewer 1, please include the verbatim recommendations on follow-up visit frequency of the corresponding guidelines for the two cohorts, i.e., before and after the implementation of the personalized discharge letter approach. This information will help readers to interpret any possible interference of guideline revision with the study intervention.

*We agree with both the reviewer and editor that further specification was needed regarding the possible guideline interference. This has been adjusted accordingly as stated in response to reviewer 1, comment 5.*
